# Walking and running in the desert ant *Cataglyphis fortis*

**DOI:** 10.1007/s00359-015-0999-2

**Published:** 2015-04-01

**Authors:** Verena Wahl, Sarah E. Pfeffer, Matthias Wittlinger

**Affiliations:** Institute of Neurobiology, University of Ulm, 89069 Ulm, Germany

**Keywords:** Desert ant, *Cataglyphis*, Stepping pattern, Inter-leg coordination, Gait

## Abstract

Path integration, although inherently error-prone, is a common navigation strategy in animals, particularly where environmental orientation cues are rare. The desert ant *Cataglyphis fortis* is a prominent example, covering large distances on foraging excursions. The stride integrator is probably the major source of path integration errors. A detailed analysis of walking behaviour in *Cataglyphis* is thus of importance for assessing possible sources of errors and potential compensation strategies. Zollikofer (J Exp Biol 192:95–106, 1994a) demonstrated consistent use of the tripod gait in *Cataglyphis*, and suggested an unexpectedly constant stride length as a possible means of reducing navigation errors. Here, we extend these studies by more detailed analyses of walking behaviour across a large range of walking speeds. Stride length increases linearly and stride amplitude of the middle legs increases slightly linearly with walking speed. An initial decrease of swing phase duration is observed at lower velocities with increasing walking speed. Then it stays constant across the behaviourally relevant range of walking speeds. Walking speed is increased by shortening of the stance phase and of the stance phase overlap. At speeds larger than 370 mms^−1^, the stride frequency levels off, the duty factor falls below 0.5, and *Cataglyphis* transitions to running with aerial phases.

**Electronic supplementary material:**

The online version of this article (doi:10.1007/s00359-015-0999-2) contains supplementary material, which is available to authorized users.

## Introduction

If you are in a North African salt pan in the middle of the day, you would probably encounter *Cataglyphis fortis* desert ants pacing around with tremendous speeds on their long legs, insects Rüdiger Wehner likes to call “race horses in the insect world” (Wehner 2009). Like race horses with their shiny and delicate bodies, they can doubtlessly exert a fascination on the observer when they attain high walking speeds while swiftly manoeuvring through their harsh environment, always on the look-out for dead insects that succumbed to the heat of the day (Wehner 1983). Individuals with a prey item, one can see running along an imaginary straight line which connects the place where they had encountered the food with the nest entrance. The kind of navigation that *Cataglyphis fortis* ants perform on their foraging excursions is an approximate form of dead reckoning, the so-called path integration where the ants are constantly connected to the nest location via an invisible link (Collett and Collett 2000; Wehner and Srinivasan 2003; Wehner and Wehner 1986, 1990). Combining path integration as a navigation mode and high walking speeds, *Cataglyphis* ants maximize their chances of finding food and returning to the nest even in the hottest times of the day without succumbing to the hostile conditions. To perform path integration *Cataglyphis* ants would need two inputs: (1) information about angles steered, that is, the direction and (2) information about the distance travelled. Directional information is provided by a celestial compass (Wehner 1982; Müller and Wehner 1988), and distance information is gained by means of a stride integrator (Ronacher and Wehner 1995; Wittlinger et al. 2006, 2007) which might be a major source of navigational errors. To better understand the stride integrator, we need a detailed analysis of walking behaviour and thus the inter-leg coordination across the entire range of walking speeds employed by *Cataglyphis fortis*. Zollikofer (1994a) demonstrated consistent use of the tripod gait in desert ants and suggested an unexpectedly constant stride length as a possible means of reducing navigation errors. During his time in Rüdiger Wehner’s lab, Christoph Zollikofer pioneered the work on walking kinematics in these fast running desert ants, and since then many details have been revealed about the locomotor behaviour of *Cataglyphis fortis* compared to other species, namely the influence of speed and curvature, of body morphology and load (Zollikofer 1988, 1994a, 1994b, 1994c) or locomotion on sloped surfaces (Seidl and Wehner 2008; Weihmann and Blickhan 2009). Nevertheless, with advanced high-speed videography at hand, we are now able to get a more thorough insight into *Cataglyphis*’ walking behaviour. Moreover, we can extend these studies not only by more detailed analyses of inter-leg coordination but also expand the range of walking speeds to where we assume its limits. The aim of this paper is to investigate the effect of walking speed on the inter-leg coordination or gait, stride length, walking speed and stride amplitude, duty factor, as well as swing and stance phase and phase relationships of all six legs.

## Materials and methods

### Ant colonies

High-speed video recordings were performed in the field near Maharès, Tunisia and in the laboratory at University of Ulm, Germany. For the laboratory recordings, several colonies of *Cataglyphis fortis* were kept and raised under annual temperature and daily light–dark cycles based on conditions in their natural habitat (20–35 °C, winter–summer; 14 h:10 h, light:dark cycle in summer). The colonies in the laboratory consisted of several hundred ants, with an active forager force of approximately 10 % of the population size. Estimated from the number of active foragers, the field colonies and the colony size were comparable. The laboratory ants received water ad libitum and were fed with honey water and insects, five times a week.

### Experimental procedure

**Fig. 1:**
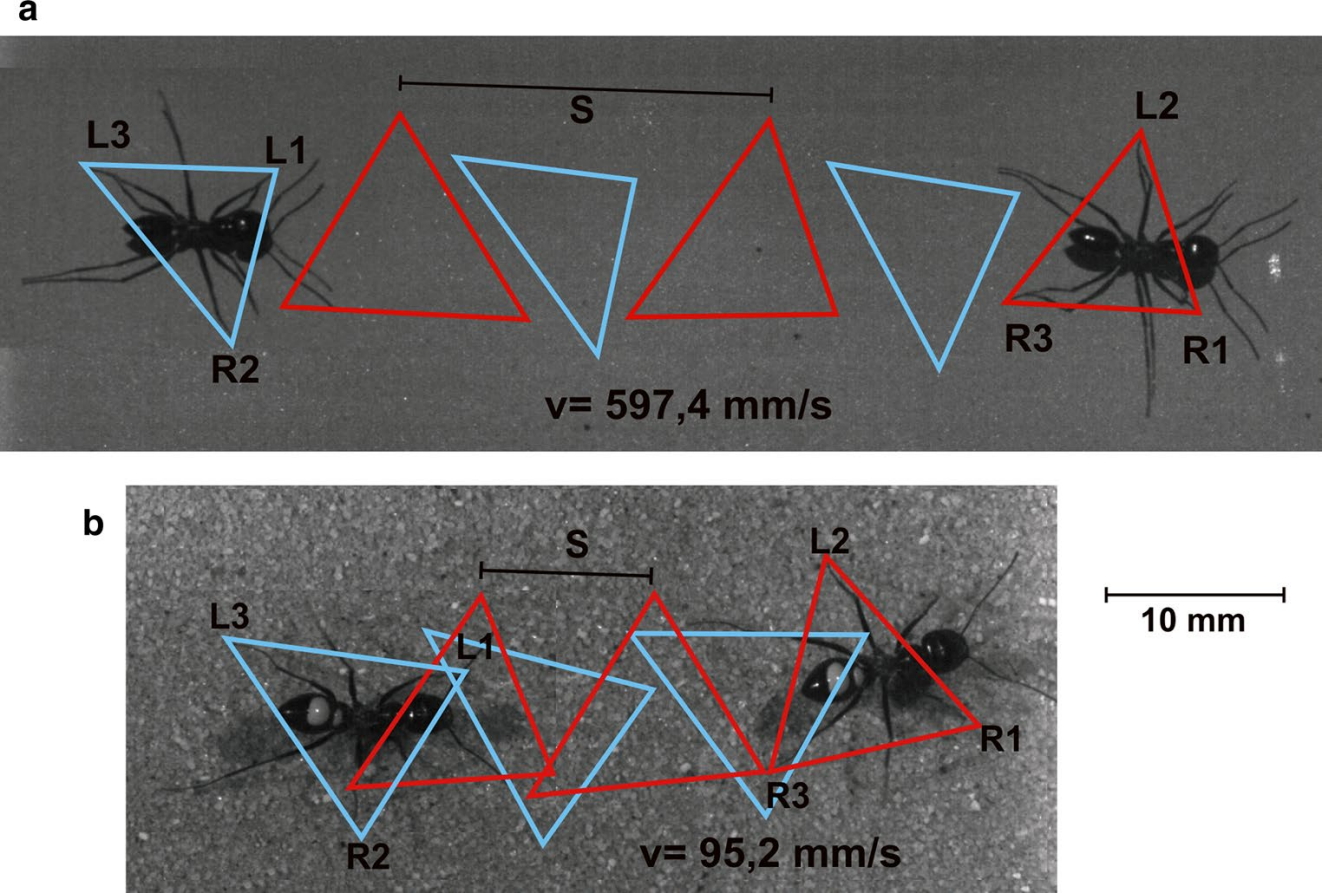
Tripod gait of a fast running and a slowly walking *Cataglyphis* individual. Six complete strides—three of each body side—captured by high-speed video are shown. Tripods formed by the right front and hind leg (R1, R3) and the left middle leg (L2) are drawn in *red*; tripods formed by the left front and hind legs (L1, L3) and right middle (R2) leg are drawn in *blue*. Stride length (*s*) was determined as the distance between two successive footfalls of the same leg. **a** Very fast running ant showing the typical tripod gait (*s* = 19.8 mm; *v* = 597.4 mm s^−1^). **b** A rather slowly walking ant also showing the typical tripod gait, however, with reduced stride length (*s* = 9.1 mm; *v* = 95.2 mm s^−1^). Single video frames of the ant, taken during the first and sixth captured steps, are pasted into the tripod analysis figure

Medium to large sized (2.5–3.3 mm alitrunk length) *Cataglyphis fortis* ants were individually marked and were filmed with a camera placed above the channel while they walked in a linear channel with a width of 7 cm and channel wall height of 7 cm. We video filmed the running ants between 0900 and 1600 h. The highest walking speeds were usually recorded around noon, when the highest air temperatures were reached in the field. Channel floors were evenly coated with a very fine layer of firmly attached white sand (0.1–0.4 mm particle size) to provide good traction and thus to facilitate slip-free natural walking and running kinematics. Film recordings were made with a high-speed camera (MotionBlitz Eosens Mini1, Mikrotron Unterschleissheim, Germany) at 500 and 1000·frames per second (Fig. 1) and shutter times of 100–200 µs. The indoor laboratory video shoots were illuminated with two fibre optic cold light sources (Schott KL 1500LCD, 150W, Schott AG, Mainz, Germany) whereas videos filmed under open sky outdoor conditions needed no external light sources since the sun provided plenty light. To get videos of very slowly walking desert ants, the channel setup was cooled down to about 10–15 °C by means of cooling pads.

### Data analysis

In the experiments, the ants walked through the channel at different speeds. Both inbound and outbound walking ants were considered for the walking analysis. Especially in the outdoor video sessions, the inbound walking ants sometimes carried a minute food item. Each individual was video recorded up to five times, consequently in the data of *N* = 388 runs up to five runs might origin from one ant. Only those individuals exhibiting regular straight and linear walks without de- or acceleration or abrupt stops were used for the tests. Each analysed walk contained at least three step cycles per leg. A 5 cm long black and white scale bar was filmed after each set of videos with the same settings to calibrate the analysed videos. Tarsal footfall positions as well as times of lift-off (or movement away from the contact point) and touch-town of the tarsal tips were digitized with ImageJ (US National Institutes of Health, Bethesda, Maryland, USA, http://imagej.nih.gov/ij/) on a frame-by-frame basis. The duration of swing phases were calculated as the difference between the time the tarsal tip lifts off the ground and subsequent touchdown of the tarsal tip of the same leg for the swing phase or vice versa for the stance phase. In the hind legs, the tarsal tip often is dragged over the floor without being lifted off the ground. Here, we define the moment when the tarsal tip leaves the contact point on the floor as start of the swing phase (Reinhardt and Blickhan 2014). The onset of stance was used as the reference time for the analysis of temporal coordination of all legs for the phase analyses. The CircStat Toolbox in MATLAB was used for phase analyses and the corresponding plots (Berens 2009; Wosnitza et al. 2013). Stride frequency is defined as the walking speed divided by the stride length. The stride length was calculated for each leg pair (L1, R1; L2, R2; L3, R3) as the mean of each leg pairs’ strides in one video sequence. Stride length is specified as the measure from two successive footfalls of the same leg of one body side (Alexander 2003). A stride should not be confused with a step, which is the distance the body covers from the footfall of a leg pairs’ left leg to the footfall of the right leg or vice versa. A stride thus basically incorporates two steps, the left and the right. Stride length is therefore actually double the step length (assuming the left step is more or less the same as the right step and walking speed is constant). When we look at the tripod shaped gait in Fig. 1, one stride describes the relationship of two successive triangles of the same colour whereas one step describes the relationship of two differently coloured successive triangles. In this account, we only employ the term stride as mentioned above and as it is defined in Alexander (Alexander 2003). The stride amplitude is a measure for the swing of one leg during a stride without the additional body movement during the swing phase (Wosnitza et al. 2013). We calculated the stride amplitude as the stride length minus swing phase duration multiplied by walking speed. The stride amplitude (Wosnitza et al. 2013) which is misleadingly called “stride length” in Hughes (1951) is technically a body coordinate based measure for the swing movement of a leg. We, however, calculated the mean stride amplitude of a run as an indirect measure from geo-coordinate based data, such as the means of stride length, swing phase duration and walking speed. We also assume a constant mean walking speed for all runs evaluated. Therefore, minor errors might occur. Although a certain variability of walking speed within a step cycle might be observed especially for the slow walks, we only evaluated video sequences with a constant mean walking speed over several step cycles. Mean walking speed was measured from the start of the first step cycle to the end of the last step cycle in one video sequence.

We calculated the tripod coordination strength (TCS) which evaluates the quality of the tripod coordination (Wosnitza et al. 2013; compare also Spagna et al. 2011). First, we calculated the time from the earliest swing onset to the latest swing termination. This gave us time *t*_1_, during which at least one of the three legs was in swing phase. Then we determined time *t*_2_, during which all three legs were in swing phase at the same time. The ratio *t*_2_/*t*_1_ then described the TCS. A TCS of 1 indicated perfect tripod coordination; it approached 0 when the temporal relationship of swing phases shifted to other coordination patterns (Wosnitza et al. 2013). The duty factor, a ratio of stance phase to cycle period can be used as a measure that describes the transition from walking to running (Alexander 2003). We measured the cycle period as the time between successive touchdowns of the same limbs. Thus, one gait cycle begins when the reference foot contacts the ground and ends with subsequent touchdown of the same foot. Since cycle period of very slow walks gets more variable and calculations of TCS or duty factors do not deliver appropriate, comparable values, we carried out a separate evaluation of walking behaviour during slow locomotion (Fig. 5). We did a frame-by-frame analysis of 76 videos within a speed range of 4.5 to 29.9 mms^−1^ (five different speed groups I–V) by classifying each frame according to its gait pattern similar to the work of Mendes et al. (2013). Each frame was assigned a certain colour and a number representing the different leg coordination types. For the different leg combinations used for our gait analysis, see supplementary material. If none of the listed leg combinations was found, the frame was classified as ‘undefined’. For each of our five speed groups, we calculated a percentage distribution of different leg combinations, which the ants applied during slow walks. Further, the frames’ gait index was averaged for each video and pooled according to the five speed groups to accomplish a more inter-individual comparison. Statistical analyses were performed with SigmaPlot 11.0 (Systat Software Inc., San Jose, California, USA). Pair-wise comparisons (Fig. 5) and comparisons of slope and *y*-intercept (Figs. 3a, 6b) were performed with a *t* test, since respective groups were all normally distributed. We fitted data with linear, power and polynomial functions and calculated *R* ^2^ in Microsoft Office Excel 2013.

## Results

The walking parameters of *Cataglyphis fortis* were evaluated spatially and temporally over the entire walking speed range from 4.5 to 619.2 mms^−1^.

**Fig. 2:**
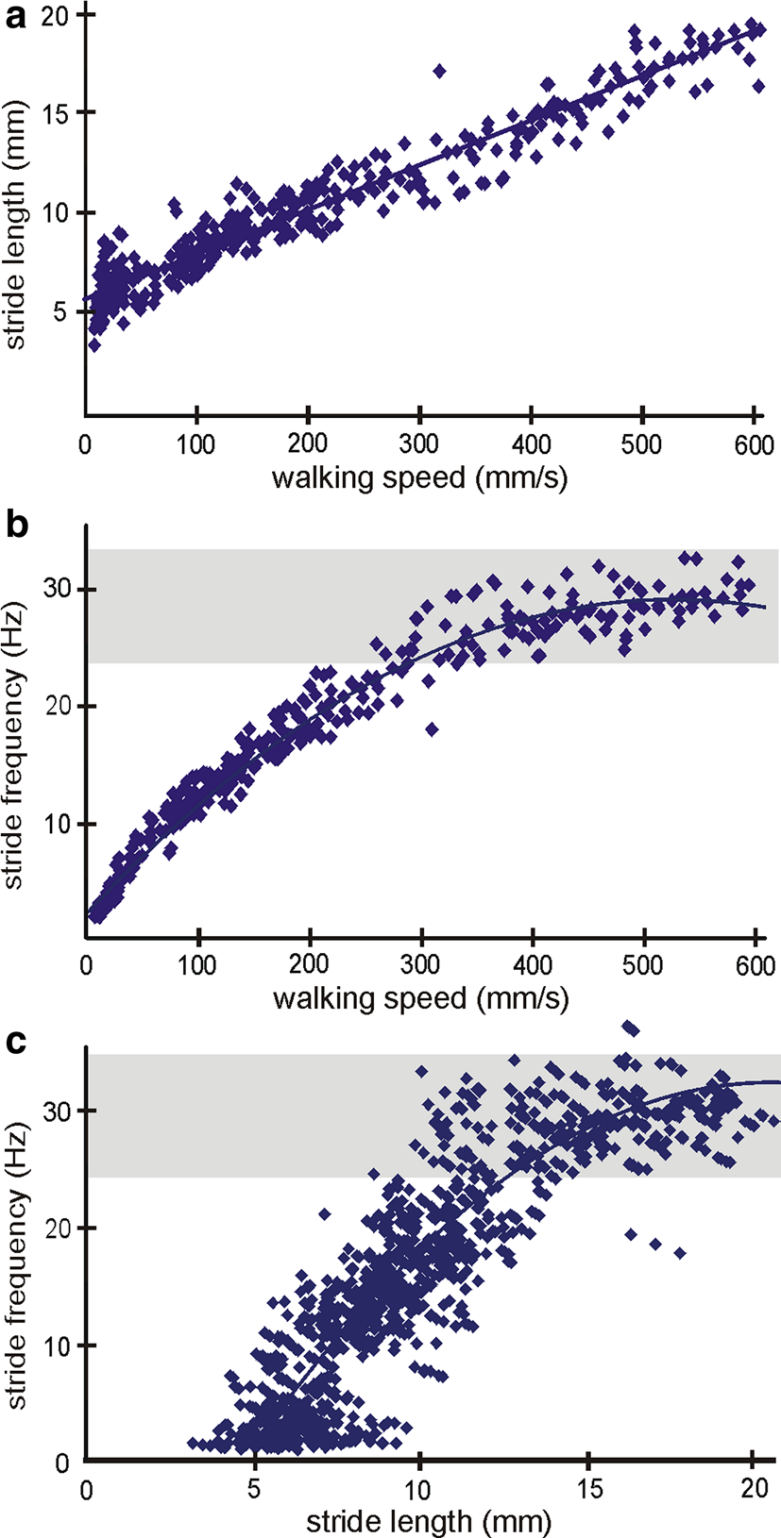
General walking parameters, stride length, stride frequency and walking speed and their relationships. Only middle leg data are plotted; *each data point* represents one video sequence (*N* = 388). **a** Stride length as a function of walking speed for the entire walking speed range. Linear regression is indicated; *y* = 0.023 × *x* + 5.93; *R* ^2^ = 0.93. **b** Stride frequency as a function of walking speed. Best fit regression is indicated; *y* = −0.0001 *x* ^2^ + 0.11*x* + 1.63; *R* ^2^ = 0.97. **c** Stride frequency as a function of stride length. Best fit regression is indicated; *y* = −0.115*x* ^2^ + 4.78*x* − 19.77; *R* ^2^ = 0.81. The *grey horizontal bar* highlights the frequency plateau (**b**, **c**)

With increasing walking speed, the stride length increases in an almost perfectly linear fashion (Fig. 2a). The faster the ant runs, the longer the strides get. The stride length increases more than fourfold over the entire speed range from 3.5 mm (at 4.5 mms^−1^) to 19.8 mm (at 589.5 mms^−1^). Stride frequency increases as a function of walking speed and levels off at a frequency plateau of around 30 Hz beginning somewhere between 300 and 400 mms^−1^ (Fig. 2b). In the desert ants, the start of the frequency plateau is a first indication that the ants attain aerial phases. Ants that are achieving longer strides, increase stride frequency to a maximum at which the frequency reaches the upper level while the strides are still getting larger. From this point on, walking speed is increased by increasing stride length only. To maximise stride length in spite of a stagnant stride frequency, the ants become “airborne” from footfall to footfall to cover a larger distance (Fig. 2c).

**Fig. 3:**
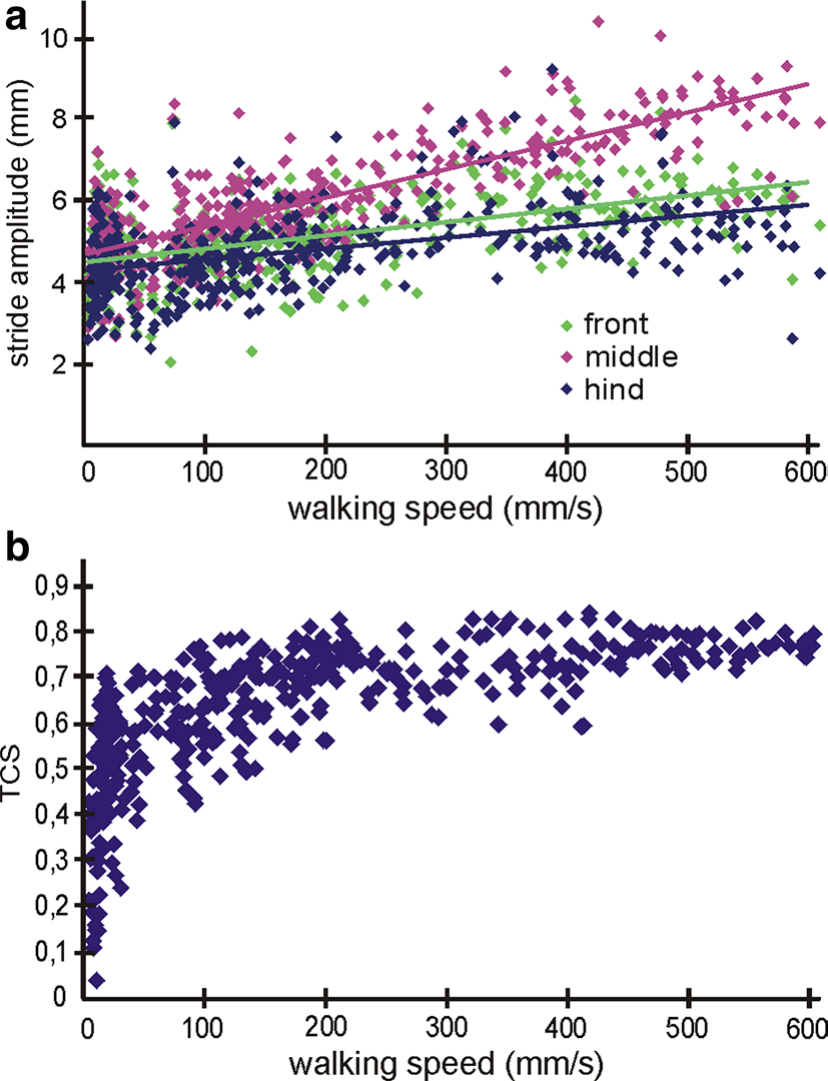
Walking parameters of *N* = 388 high-speed videos. **a** Stride amplitude as a function of walking speed for all three leg pairs. Leg pairs are represented in *green* (front legs), *magenta* (middle legs) and *blue lines* (hind legs); linear regression lines are indicated, front legs: *y* = 0.0032 × *x* + 4.54; *R* ^2^ = 0.28; middle legs: *y* = 0.0067 × *x* + 4.72; *R* ^2^ = 0.66; hind legs: *y* = 0.0026 × *x* + 4.33; *R* ^2^ = 0.20. The slope of front and middle legs differ significantly (*t* test, *p* < 0.05) as well as that of the middle and the hind legs (*t* test, *p* < 0.05) while front and hind legs are not significantly different. For all leg pair combinations, the *y*-intercept is significantly different (*t* test, *p* < 0.05). **b** Tripod coordination strength (TCS, for definition see “Materials and methods”) as a function of walking speed

The stride amplitude (Wosnitza et al. 2013), is a body coordinate based measure for the swing of a leg. The stride amplitude of the middle leg shows a good linear correlation with increasing walking speed. The amplitude of the middle legs doubles, whereas the amplitudes of front and hind legs do not increase significantly and show only a weak correlation (*R* ^2^ = 0.28, front legs; *R* ^2^ = 0.66, middle legs; *R* ^2^ = 0.20, hind legs) (compare Fig. 3a). For the middle leg, this means that 66 % of the variability can be described by the linear regression model.

**Fig. 4:**
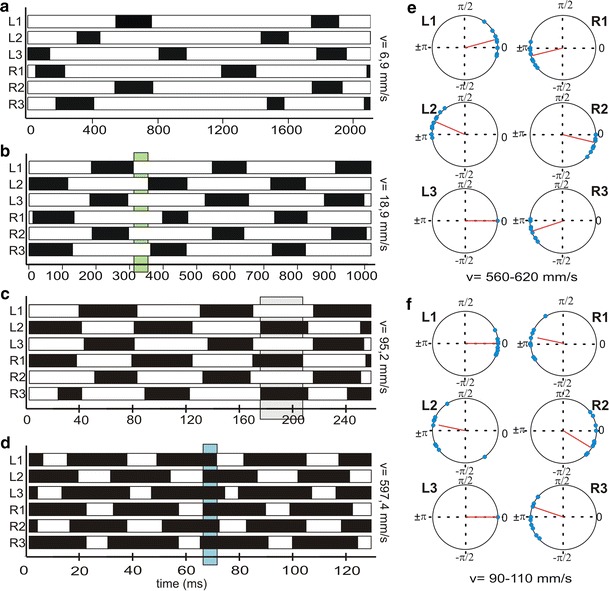
Analysis of inter-leg coordination. (**a**–**d**) Footfall patterns, podograms, of all six legs from four runs with different walking speeds, from minimum to almost maximum speed. *White bars* represent stance phases, *black bars* represent swing phases; *L* left, *R* right body side; *1*, *2* and *3*, front-, mid- and hind leg. *Shaded areas* highlight exemplary tripod gait patterns with stance phase overlap (*green*, see **b**) and swing phase overlap (*blue*, compare **d**). *Shaded area* (*grey*, compare **c**) highlights an exemplary footfall pattern with a TCS of 0.77. Walking speeds are 6.9 mms^−1^ (**a**), 18.9 mms^−1^ (**b**), 95.2 mms^−1^ (**c**) and 597.4 mms^−1^ (**d**). (**e**, **f**) Phase plots of the stance, phase onset of all legs with respect to the left hind leg; *L1*, *L2*, *L3*, left side front, middle and hind leg; *R1*, *R2*, *R3* right side front, middle and hind leg. Two exemplary walking speed ranges are shown, 560–620 mms^−1^ (**e**) and 90–110 mms^−1^ (**f**). *Blue* data from five runs; *red line* mean vector

**Fig. 5:**
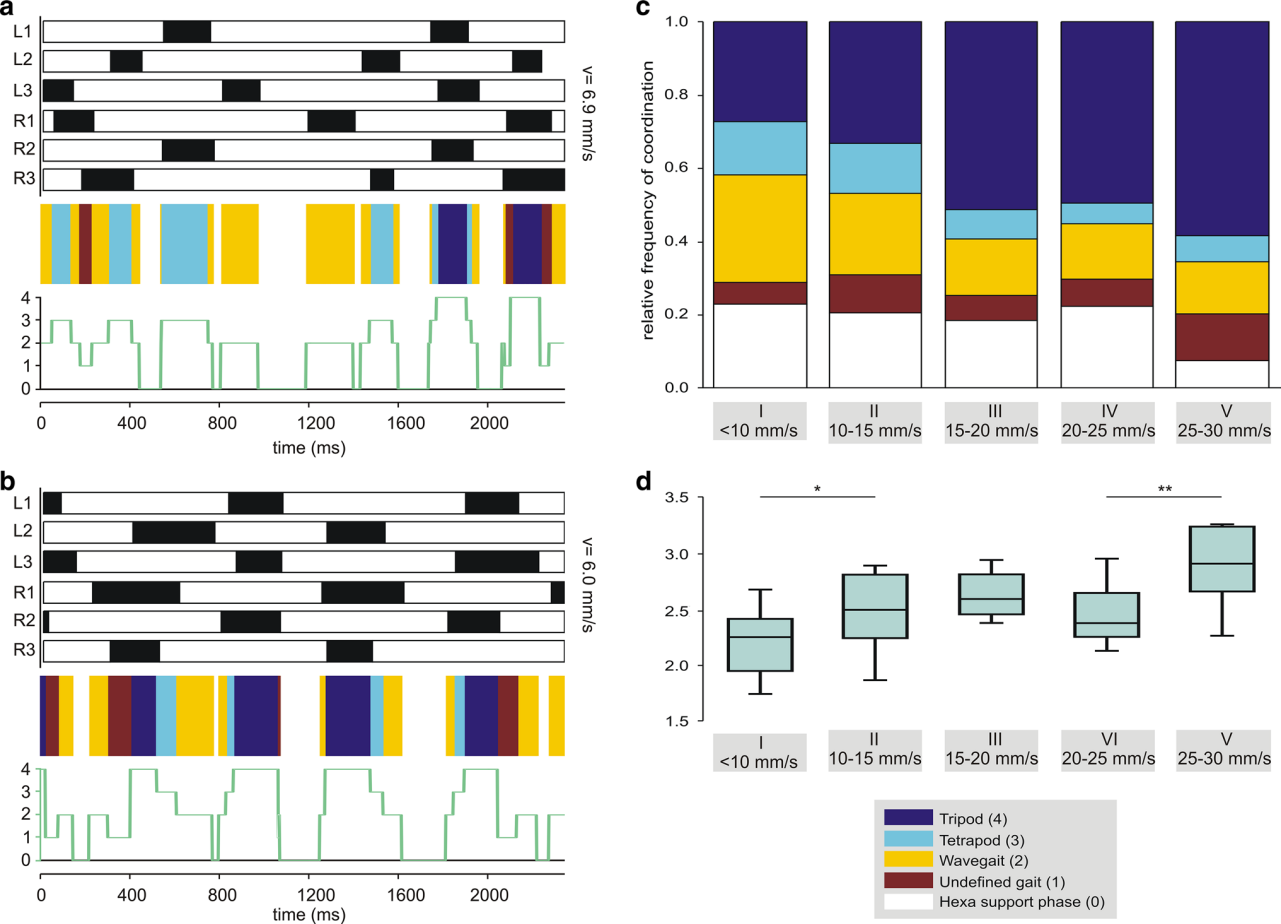
Quantification of gait pattern during slow walking. Gait Pattern analysis for ants walking at **a** 6898 mms^−1^ and **b** 5959 mms^−1^. (**a**, **b**) Podogram (*above*), coloured coding (*middle*) and indexing (*below*). Illustration details of the podograms as in Fig. 4. For the colour coding and the indexing we used five different classifications: ‘tripod’ (*dark-blue*, 4), ‘tetrapod’ (*light-blue*, 3), ‘wavegait’ (*yellow*, 2) or ‘hexa support phase’ (*white*, 0). If none of these possibilities were applicable, the frame was classified as ‘undefined’ (*red*, 1). For the list of exact leg combinations representing a typical gait see supplementary material. **c** Quantification of the *N* = 76 slow walking speed videos were grouped into five categories: *I* 5–10 mms^−1^ (17 videos, 8950 frames; 27, 14, 29, 6, 23 %), *II* 10–15 mms^−1^ (16 videos, 7376 frames; 33, 14, 22, 10, 21 %), *III* 15–20 mms^−1^ (20 videos, 7116 frames, 51, 8, 15, 6, 18 %), *IV* 20–25 mms^−1^ (14 videos, 4284 frames; *V* 25–30 mms^−1^ (9 videos, 2423 frames; 58, 7, 15, 13, 7 %). The percentage information in *brackets* after the semicolon is rounded and is arranged as follows: tripod, tetrapod, wavegait, undefined gait, hexa support phase. **d** The averaged index for each video provide a more individual analysis of the ants’ walk. Group I differs significantly from group II (*t* test; *p* = 0,014); the same was true for group IV and group V (*t* test; *p* = 0,004). The three intermediate speed groups (II, III, IV) do not show any statistically significant differences to their respective neighbouring groups

Since *Cataglyphis* is known to employ tripod coordination over most of the walking speed range (Seidl and Wehner 2008; Wittlinger et al. 2006, 2007; Zollikofer 1988, 1994a), we evaluated the quality and synchrony of the tripods by means of the tripod coordination strength (TCS) (Wosnitza et al. 2013; compare also Spagna et al. 2011). The variability of the TCS decreases with increasing walking speed and at the same time converges towards the maximum levels of around 0.7 to 0.85. From a walking speed of around 300 mms^−1^, the variability is least whereas at lower speeds, the TCS varies between 0.02 and 0.78. Above walking speeds of around 300 mms^−1^, *t*_2_ and *t*_1_ of the TCS both remain at constant levels of 12–22 ms (*t*_2_) and 24–34 ms (*t*_1_). To further analyse the inter-leg coordination and the phase relationships of the tripods, we made footfall patterns or podograms that show the swing and stance phases of every leg as black (swing) and white (stance) bars along a timeline (Fig. 4a–d). The podogram in Fig. 4a shows a very slow locomotion. This walk with 6.9 mms^−1^ is at the lower edge of walking speed and exemplifies that calculations used for the walking speed larger than 30 mms^−1^ (e.g. TCS and duty factor) do not provide any useful information in this case. Therefore, slow walks were analysed and quantified separately in Fig. 5. In contrast, the podograms of the higher walking speeds (Fig. 4b–d) beautifully show tripod coordination. The green bar in Fig. 4b highlights the stance phase overlap where all six legs are on the ground at the same time (hexa support phase) for a relatively slow walk. The blue bar in a very fast run (Fig. 4d), however, exemplifies the swing phase overlap (aerial phase) which is the time where the ant is airborne—all legs lost ground contact—except for some cases where the hind legs might be dragged over the substrate. We also calculated phase plots of the stance phase onset of all six legs with respect to the left hind leg (Fig. 4e, f). Each of the three leg pairs shows an antiphasic relation. The legs are more or less coordinated as a tripod of L1, L3, R2 and L2, R1, R3. Figure 4e and f show that the middle leg of one tripod tends to touchdown first, and then the hind leg touches the ground, followed immediately by the front leg, which is nearly in phase with the hind leg. The data points (blue) of slow walks (Fig. 4f) are more spread than in the fast walks (Fig. 4e). This also confirms what we already know from the TCS analysis. The tripods are never perfectly in phase and the TCS improves with increasing walking speed. Nevertheless, we can see how a tripod is temporally formed. The three legs of one tripod never touch down or lift-off the ground simultaneously but the temporal coordination improves with increasing walking speed.

In a separate analysis, we focused on walking behaviour during slow locomotion below walking speeds of 30 mms^−1^. A continuous gait transition from tripod to tetrapod to wavegait coordination is proposed for hexapods with decreasing walking speeds (Schilling et al. 2013). Throughout its entire walking speed range, *Cataglyphis fortis* ants predominantly walk in tripod-fashion, which is also true for the runs at the lower edge of walking speeds (Fig. 5b). However, it seems evident that with decreasing speed, the tripod coordination is getting more inconsistent and the number as well as the proportion of other stepping patterns increases. We observed that ants use poorly coordinated or non-tripod pattern only for a short period of time. Almost all ants that show tetrapod, wavegait or other undefined stepping patterns during more than one step cycle, subsequently display the transition into tripod coordination within the same video sequence (Fig. 5a).

To illustrate the variability of leg coordination of very slow walks, we not only used the podograms but also colour coding and indexing of stepping patterns (see examples in Fig. 5a, 6.9 mms^−1^ with the transition from tetrapod to tripod coordination; and Fig. 5b, 6.0 mms^−1^ with tripod coordination). The colour coding and indexing was also applied to quantify the leg coordination in all (*N* = 76) videos below walking speeds of 30 mms^−1^. In Fig. 5c, we give a summary of percentage values of different gait patterns. They show that with increasing speed, the proportion of tripod gait increases, while tetrapod coordination and wave gait decreases as well as the time where all six legs have ground contact simultaneously (hexa support phase). To further compare the individual performance, we averaged the index that was assigned to each frame in one video (Fig. 5d). This shows that with increasing speed the indices also increase, which reflects the increasing consistency of the tripod.

Note that a large fraction of non-tripod combinations forms in the transitional time from one tripod group (e.g. L1, R2, L3) to the subsequent one (L2, R1, L3). When we look at Fig. 5b, we clearly notice tripod coordination in the podogram, though other coordinations are also present to a large extent (compare Fig. 5b, colour coding and indexing graph). Hence, our analysis shows that even slow walking *Cataglyphis* ants preferentially employ tripod coordination, but with decreasing speed, the tripod gets more variable and other leg coordination are used as well.

**Fig. 6:**
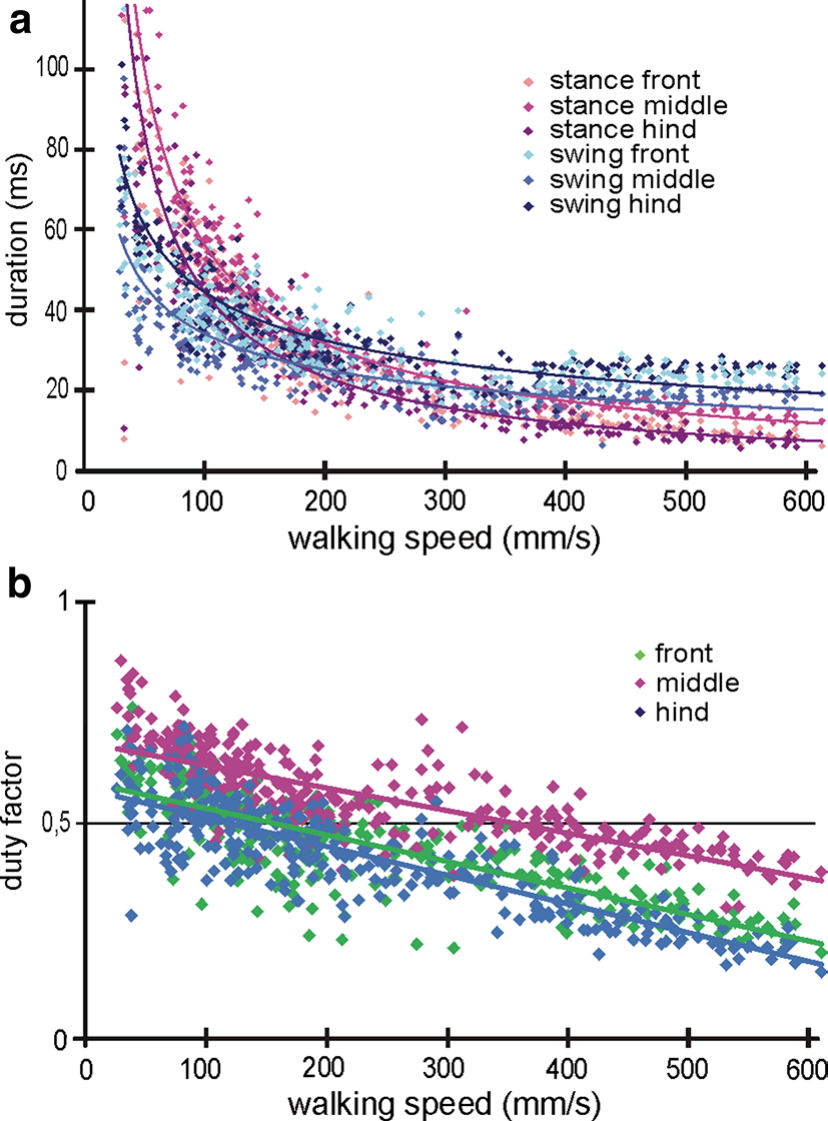
Stance and swing phase duration and duty factor. **a** Durations of stance (*three shades of purple*) and swing phases (*three shades of blue*) as a function of speed of all three leg pairs. Graphical fits are represented for middle and hind legs in *purple* (*y* = 0.22*x* ^−0.38^, *R* ^2^ = 0.75; *y* = 0.26*x* ^−0.41^, *R* ^2^ = 0.77) and *blue lines* (*y* = 1.76*x* ^−0.83^, *R* ^2^ = 0.88; *y* = 1.58*x* ^−0.79^, *R* ^2^ = 0.84), respectively. Runs without tripod coordination with walking speeds below 25 mms^−1^ have not been considered for these graphs. **b** Duty factor, which is the ratio of stance phase duration to duty cycle, versus walking speed for all three leg pairs. Leg pairs are represented in *green* (front legs), *magenta* (middle legs) and *blue lines* (hind legs); linear regression lines are indicated, front legs: *y* = −0.0005*x* + 0.59; *R* ^2^ = 0.66; middle legs: *y* = −0.0004*x* + 0.66; *R* ^2^ = 0.61; hind legs: *y* = −0.0006*x* + 0.57; *R* ^2^ = 0.73. The slopes of front and middle legs differ significantly (*t* test, *p* < 0.05) as well as that of the middle and the hind legs (*t* test, *p* < 0.05) while front and hind legs are not significantly different. For all leg pair combinations, the *y*-intercept is significantly different (*t* test, *p* < 0.05)

We will now have a look at the swing and stance phase durations as a function of walking speed (Fig. 6a). Both the swing phases and the stance phase are significantly reduced at the initial part of the walking speed range. While the stance phases are longer than the swing phases at lower walking speeds, this relation reverses at higher walking speeds. Interestingly, the reversal in the hind legs and front legs occur much earlier (hind legs: 95 mms^−1^) than in the middle legs (middle legs: 349 mms^−1^). The duration of swing and stance phases in *Cataglyphis* decrease with increasing walking speed in the fashion of a power function (compare Fig. 6a) and remains more or less constant from a walking speed of 300 mms^−1^ in (Fig. 6a). For a large part of the range, the walking speed is increased by reducing the stance phase while the swing phase stays rather constant. At highest walking speeds, the middle legs have the shortest swing phase and longest stance phase of all legs. Hence, the middle legs are the first to touch the ground and the last to lift-off again. We define the swing phase as the time where the leg is in motion, that is, the time from where the tarsal tip of one leg leaves the contact point on the substrate to the subsequent contact point on the ground. The hind legs displayed a peculiarity in that they often moved the tarsi along the floor without being lifted off the floor. This “gliding phase” is part of the swing phase, although the gliding hind legs that are basically dragged behind the ants still touched the ground. This phenomenon has recently been observed in *Formica* ants as well where the tarsi of the hind legs were regularly dragged over the substrate without being significantly raised off the ground (Reinhardt and Blickhan 2014). In some video sequences, we were able to observe that the tarsal claws were retracted before the gliding phase and thus the swing phase started.

Another measure for the phase relationship is the duty factor. Besides, it is one measure that characterises the dynamics of when the transition from walking to running occurs. It is assumed that at values of around 0.5, this transition happens (Alexander 2003). With increasing walking speed, the duty factor decreases linearly for all three leg pairs. The hind legs are the first to fall below the duty factor of 0.5 at 132 mms^−1^, then the front legs (at 182 mms^−1^) followed by the middle legs (at 369 mms^−1^). The middle legs are the last to reach aerial phases and thus determine the walking speed threshold at which the transition from walking to running occurs. From that speed on the ants are “jumping” from step to step to further increase their strides (compare the gaps between the triangles in Fig. 1a).

## Discussion

In 1850, the long legged desert ant of the genus *Cataglyphis* was described as a “most remarkable appearance within the insect fauna of old world desert areas” (Foerster 1850). With its long legs that characterise all *Cataglyphis* species, *Cataglyphis fortis* reaches the highest running speeds with values of up to 0.7 ms^−1^ in the literature (Wehner 1983). How are these ants able to reach such high running speeds? This question was already tackled by Christop Zollikofer when he was a PhD student in Rüdiger Wehner’s lab (Zollikofer 1988, 1994a, 1994b, 1994c). His work was the beginning and basis of our data collections and analyses that we present here. With more advanced techniques, we were able to expand the range of walking speeds to its limits and to extend the analysed parameters.

### Contribution and role of the leg pairs to locomotion

The variation in stride amplitude as well as in stance and swing phase duration of the front legs tends to be higher than that of the middle and hind legs. A freer and unhampered positioning of the frontal tarsi is possible here because there are no legs in front of them with which they could interfere and thus limit their range in frontal direction. One could assume that the front legs generate the smallest forces with reference to the body movement. In *Acheta domestica* (Harris and Chiradella 1980), *Carausius morosus* (Cruse 1976) and *Periplaneta americana* (Full and Tu 1991) force measurements confirm the front leg part in keeping the body’s stability. The longitudinal forces of the protarsi act against the moving direction.

Interestingly, Zollikofer (1988) observed a higher correlation of the front leg stride length with walking speed than that of the middle and hind legs. Moreover, he describes that when sprinting, the front legs of *Cataglyphis bombycina* specimens would often not leave any tarsal imprints on the smoked-glass plates that he used for the stride analysis. This fact made him conclude that at very high running speeds, the ant’s front legs would stop touching the ground, performing a form of quadrupedalism (Zollikofer 1994b). Loss of ground contact is well known in insects (*Periplaneta americana*: Full and Tu 1991), in crabs (*Ocypode quadrata*: Blickhan and Full 1987) and in vertebrates (Heglund et al. 1974).We cannot confirm this observation in *Cataglyphis fortis*, although we analysed a large number of runs from the laboratory and the field over the entire speed range. Sometimes, however, when ants got startled, they showed a short sequence where they accelerated, rising the head and prosoma and lifted the forelegs off the ground. They performed a movement comparable to a “wheelie” known from motorbikes when their front wheel loses ground contact during high accelerations. However, we did not see this behaviour in fast running ants with constant speed.

The middle legs seem to play a distinctive role in the locomotor apparatus of *Cataglyphis* *fortis* desert ants. They show the longest stance phase and the shortest swing phase of all legs. The middle leg of the tripod is thus the first leg touching down and the last lifting off the ground. Hence, the duty factor of the middle legs is the last to underscore 0.5 with increasing speed and thus determines the start of aerial phases. At high running speeds, the tarsi of the middle legs show the most distal trace of swing and are positioned at a great lateral distance reaching over the neighbouring legs without interfering with them (Zollikofer 1988). Although this overlap happens, the legs are not hampering each other. Further, the middle legs also perform the largest stride amplitude. Considering all this, we may conclude that the middle legs exert the biggest influence on the speed and thus on locomotion.

The stance phase of the hind legs at high walking speeds is very short compared to that of the other legs. This might be due to the fact that the hind legs display something like an intermediate phase where the tarsi are moved along the floor without being lifted off the ground. This gliding phase is a part of the swing phase, although the gliding hind legs that are basically dragged behind the ants’ body probably still provide support and thus stability, while they are already swung. This phenomenon has also been recently observed in spiders and Formica ants (Spagna et al. 2011; Reinhardt and Blickhan 2014). Moll et al. (2013) also present an example of a grass-cutting ant that gains static stability by sliding hind legs during transport of load.

### Stepping pattern of slow and fast walking ants

Leg coordination during locomotion is flexible and can be adapted according to environmental circumstances (Alexander 1989). Walking speed can be one of those factors modulating locomotor output. With changes in walking speed quadrupeds, like horses, adapt their leg coordination to achieve an energetically optimal locomotion (Hoyt and Taylor 1981). Thereby, the transition from one to the next gait occurs in a discontinuous way. In hexapods also different gait types are known, but the question of gait transition has not yet been resolved (Graham 1985; Mendes et al. 2013). After examinations in several species, the current understanding is that the different leg patterns are part of a continuum with a continuous transition from tripod to tetrapod to wavegait coordination with decreasing walking speed (Schilling et al. 2013).

Stick insects (*Carausius morosus*) have been observed to use tetrapod coordination during slow locomotion but switch to tripod pattern with higher speeds (Wendler 1964; Graham 1972, 1985). The analysis of kinematics and walking behaviour in cockroaches (*Periplaneta americana* and *Blaberus discoidalis*) revealed two different types of tripods for locomotion, a low-speed amble and a high-speed trot (Delcomyn 1971; Bender et al. 2011). Fruit flies (*Drosophila melanogaster*) prefer tripod gait during the entire range of walking speeds, but leg coordination also gets more variable with the decrease in walking speed (Strauss and Heisenberg 1990; Mendes et al. 2013; Wosnitza et al. 2013). Wood ants (*Formica polyctena*) show stable tripod coordination during the entire range of running speed (Reinhardt and Blickhan 2014).

Our results show that the walking behaviour of desert ants (*Cataglyphis fortis*) is in close agreement with that described in *Drosophila melanogaster* and *Formica polyctena*. Desert ants employ tripod gait as their major coordination pattern over the entire walking speed. This was also the case for very slow walks, where tripod pattern was generally preserved. However, it also becomes apparent that during slow walks, synchrony of tripod coordination could be reduced or other non-tripod combinations, especially tetrapod coordination could occur, as well. This variability shows that *Cataglyphis fortis* does not need to rely strictly on tripod coordination and is *per se* able to use different patterns during walking.

However, the still preferred use of tripod seems to be kind of advantageous, probably it is an option to reduce errors arising from the iterative processes of path integration. The preference of tripod coordination also during slow walks shows that *Cataglyphis* ants mostly remain at the upper end of gait continuum proposed for hexapods (see explanation above). Regarding the higher variability of leg coordination during slow locomotion, ants scale down slightly from this upper end. It is conceivable that ants might also be able to reach the lower part of the continuum, yet in our investigation this was never evident.

The very slow walks rarely occur in the field. We know from observations that the walking speed employed during foraging is reached within the first two strides. To make the ants constantly walk below 30 mms^−1^ speed, we had to chill the environment, which in this case was a walking channel in the laboratory. Very rarely did we observe ants in the field in late spring and on relatively chilly early mornings walking at very low speeds out of the nest and soon back into the nest. They have never been observed to forage under these chilly conditions.

The quality of tripod coordination can be evaluated by means of a simple measure of tripod coordination strength (TCS) (Fig. 3b) (Wosnitza et al. 2013; compare also Spagna et al. 2011). With increasing walking speed, the TCS reaches values above 0.7 but never goes beyond 0.85. The legs of one tripod are at a minimum 15 % out of phase, even at highest walking speeds with maximum stride frequencies. From a walking speed of around 300 mms^−1^ on *t*_2_ and *t*_1_ of the TCS, both remain at a constant level of 12–22 ms (*t*_2_) and 24–34 ms (*t*_1_). This corresponds to the swing and stance durations that remain relatively constant for these higher speeds (see Fig. 6a). A TCS of 1.0 might increase the chance of jerky movements concentrating all impact forces of one tripod into one instant; especially at high speeds, there are less than 18 ms to distribute all ground reaction forces over the contact phase (compare Fig. 6a). As a result, a slight cutback of the TCS still assures a smooth run with maximum stability. The ants reach a TCS larger than 0.5 (an overlap of at least 50 %) from very low walking speeds on, while TCS smaller than 0.5 only occurs at walking speeds below 100 mms^−1^. If we compare TCS of *Cataglyphis* and *Drosophila* which can be between 0.1 and 0.8 (Wosnitza et al. 2013), we find that *Drosophila* at top speeds displays TCS comparable to *Cataglyphis*. Due to the wide range of walking speeds, *Cataglyphis* reaches top TCS values already at one-fifth of its speed range. The ants never touch ground with the tarsi associated with one tripod at the same time but kind of unroll the tripod like a ‘functional foot’ tarsal claw after tarsal claw. Especially at high walking speeds, the legs seem to act in a specific sequence. This tendency was also observed in *Drosophila* (Wosnitza et al. 2013). The alternating tripods are comparable to the alternating footfalls of bipedal walking animals (Full and Tu 1991). The big difference, however, is that tripods engage a larger area and thus provide more static stability especially for slower walking speeds whereas at higher walking speeds static stability is replaced by dynamic stability (Ting et al. 1994; Zollikofer 1994c).

### How do *Cataglyphis* ants reach high running speeds?

Stride frequency increases in a nonlinear fashion with increasing walking speeds. The stride frequency levels off at around 30 Hz and shows a frequency plateau. From this point on, walking speed is increased by increasing stride length only. Heglund et al. (1974) described that a constant stride frequency can be an indicator for a change in gait. Small animals reach a certain speed with smaller strides and higher stride frequencies (Heglund et al. 1974; Zollikofer 1988). In the desert ants, the start of the frequency plateau is a first indication that the ants attain aerial phases. Zollikofer already presumed a frequency plateau for *Cataglyphis,* although he did not observe one. With maximum frequencies of 28 Hz, the plateau was not yet evident (Zollikofer 1988).

Aerial phases during running are also known from cockroaches (Full and Tu 1990, 1991) and vertebrates (Heglund et al. 1974). However, this is not necessarily true for all animals. For instance, ghost crabs, wood ants, ostriches, cockroaches and the American wandering spider can reach a frequency plateau without aerial phases by means of compliant legs and the employment of grounded running (compare Blickhan and Full 1987; Reinhardt and Blickhan 2014; Rubenson et al. 2004; Ting et al. 1994; Weihmann 2013). The difference is probably due to the relatively longer legs of *Cataglyphis*, which changes the biomechanics of walking. Longer legs mean larger strides in terms of stride amplitude and stride length. This characterizes the desert ants as stride length maximizers (Zollikofer 1988).

The duty factor, a ratio of stance phase to cycle period, is a measure that describes the transition from walking to running (Alexander 1984, 2003). At values below 0.5, swing phases are longer than stance phases, and thus aerial phases occur. Horses, dogs, ostriches and lizards reach duty factors well below 0.5 (Alexander 1984; Fieler and Jayne 1998). Cockroaches as fast-running specimens in the insect world, however, rarely reach such small duty factors (Ting et al. 1994). The middle legs of *Cataglyphis fortis* are the last of the three leg pairs to fall below the duty factor of 0.5 at a speed of 369 mms^−1^ (compare Fig. 6b). At speeds between 132 and 369 mms^−1^, the ants are in a kind of transitional phase where the front and middle legs are already showing aerial phases while at least one middle leg has still ground contact. The gait transition is not abrupt at all, which means that the ants probably adopt a kind of grounded running within quite a wide range of running speeds. Thus, the dynamics of *Cataglyphis fortis*’ locomotor apparatus seems to be quite similar to those of *Formica* worker ants and even similar to birds, but distinctively different from those of human beings (compare Reinhardt and Blickhan 2014; Rubenson et al. 2004).

In several insect species (Wilson 1966; Graham 1972; Strauss and Heisenberg 1990), stance phase duration becomes shorter with increasing speed, while swing phase duration remains largely constant; at the fastest speeds, the durations of both swing and stance phases equalize (Mendes et al. 2013; Wosnitza et al. 2013). The duration of swing and stance phases in *Cataglyphis* decreases with increasing walking speed and remain more or less constant at the upper end of the range (Fig. 6a). This corresponds approximately with the observations Delcomyn made in *Periplaneta americana* (Delcomyn 1971). In his observations, the swing and stance phases are reduced at low stride frequencies. While in *Cataglyphis* at lower speeds, stance phases are longer than swing phases, at high walking speeds the swing phases are longer than the stance phases. This reversal occurs for the hind legs already at around 95 mms^−1^, and for the middle legs only at much higher speeds of 349 mms^−1^. The walking speed (from 200 mms^−1^ on) is increased by reducing stance phase while the swing phase stays rather constant.

Walking speeds of up to 0.7 ms^−1^ have been reported for *Cataglyphis* *fortis* (Wehner 1983). Although we video filmed in the field several times at optimal conditions, we never measured higher walking speeds than 0.62 ms^−1^. We believe that this is the upper limit of walking speeds for *Cataglyphis fortis* ants in the field site near Maharès, coastal Tunisia, which admittedly never reaches such temperature extremes like for instance the Chott El Cherid in central Tunisia.

Why is fast running important anyway? Fast running helps the ants to quickly cover large areas and thus to enhance the chance of finding food and then back home. It is probably also advantageous with regard to potential danger coming from predators and enemies like robber flies, spiders, fringe toe lizards and conspecific ants (Dahbi et al. 2008; Schmid-Hempel and Schmid-Hempel 1984; Wehner et al. 1992). Hence, the ants reduce the time they are exposed to their harsh habitat. Long legs do not only help to reach larger strides and thus high walking speeds. They can also help to minimize heat stress (Zollikofer 1994b). Even slightly above the hot desert floor, temperatures decrease to values that the ants still can tolerate (Zollikofer 1988; Wehner et al. 1992; Gehring and Wehner 1995).

### Outlook

It seems that every pair of leg contributes in a distinctive way to the ants’ locomotion. The middle legs seem to play a major role in gaining speed and the hind legs contribute in supporting stability. Nevertheless, ground reaction force measurement of the legs would be desirable to further confirm our conclusions. With higher walking speed, the stride frequency levels off and *Cataglyphis fortis* ants show aerial phases to expand the walking speed range. Each tripod group is used as a functional foot literally jumping from footfall to footfall comparable to our human run. Consistent tripod coordination throughout the entire walking speed range may be advantageous for the stride integrator. The occurrence of very slow walking speeds, where the non-tripod stepping patterns are mostly observed is usually restricted to walks inside the nest and the immediate surroundings of the nest entrance. Especially on foraging excursions, where higher walking speeds occur—never below 30 mms^−1^—robust and steady stepping coordination might induce errors as minimal as possible.

## Electronic supplementary material

Below is the link to the electronic supplementary material.

Supplementary material 1 (DOCX 166 kb)
