# Supplementary material for: Walking and running in the desert ant Cataglyphis fortis
Source: J Comp Physiol A Neuroethol Sens Neural Behav Physiol. 2015 Apr 1;201(6):645–56. doi: 10.1007/s00359-015-0999-2 (PMC4439428; doi:10.1007/s00359-015-0999-2)
Supplement: Supplementary file 1 — Supplementary material 1 (DOCX 166 kb) [file 359_2015_999_MOESM1_ESM.docx]

Journal of Comparative Physiology A

**Walking and running in the desert ant Cataglyphis fortis.**

Verena Wahl, Sarah Pfeffer, Matthias Wittlinger

Institute of Neurobiology, University of Ulm, D-89069 Ulm, Germany

Corresponding author: Matthias Wittlinger

[Matthias.wittlinger@uni-ulm.de](mailto:Matthias.wittlinger@uni-ulm.de)

Institute for Neurobiology

Helmholtzstraße 10/1

University Ulm

89081 Ulm


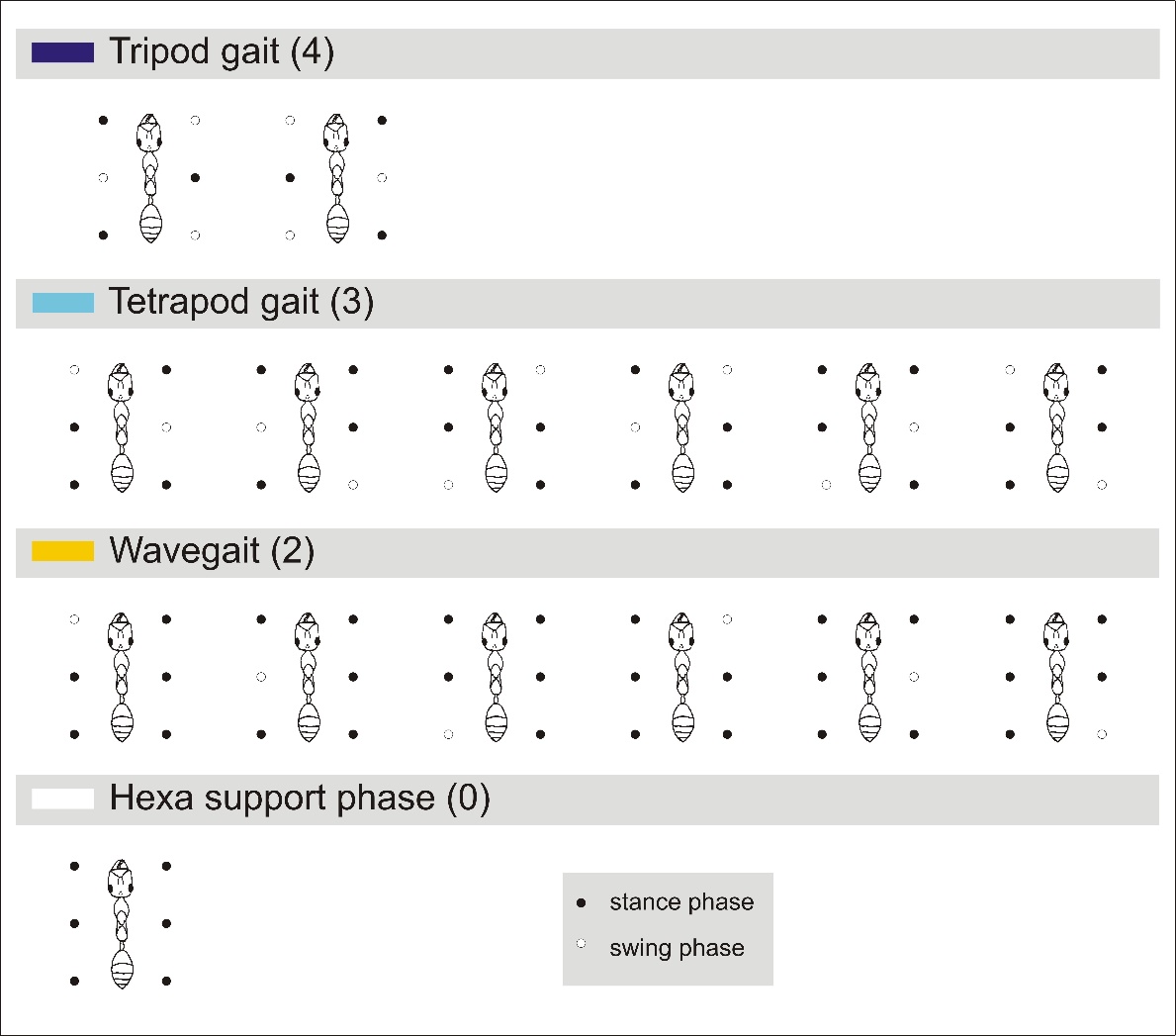


Supplementary Figure.

Leg combinations used for respective gait patterns.

The classification of different leg combinations were used for the colour coding and the indexing to quantify slow walking ants according to their used gait patterns. The analysis was similar to the quantification of gait parameters in *Drosophila melanogaster* of Mendes (Mendes et al. 2013, see their figure 4). But in contrast to this work we used more categories to describe the ants’ walks. We added ‘wavegait’ and ‘hexa support phase’ to our analysis. This is in our case reasonable since we looked at the lower range of walking speeds. Locomotion within the speed range of 4.5 mm s^-1^ to 29.9 mms^-1^ gets more variable as well as slower and therefore the number of frames showing wavegait and frames where all six legs have ground contact increase considerably.

In ‘tripod gait’ three legs are in swing phase at once, while the other three are in stance phase. One tripod contains the fore and hind leg of one side and the middle leg of the contralateral side. In ‘tetrapod gait’ two legs are in swing phase, while the other four are in stance phase. The lifted or moved legs must be on the contralateral side but not from the same leg pair. In ‘wavegait’ one leg is in swing phase, while the other five are in stance phase. In the category of ‘hexa support phase’ no leg was in swing phase, but all in stance phase. All legs had ground contact, tarsal claws not moving.
